# The role of the built environment and private rooms for reducing central line-associated bloodstream infections

**DOI:** 10.1371/journal.pone.0201002

**Published:** 2018-07-27

**Authors:** Liam O’Neill, Sae-Hwan Park, Frank Rosinia

**Affiliations:** 1 Department of Health Behavior and Health Systems, School of Public Health, University of North Texas-Health Science Center, Fort Worth, Texas, United States; 2 Department of Anesthesiology, John Peter Smith Hospital, Fort Worth, Texas, United States; Medical University Graz, AUSTRIA

## Abstract

Private hospital rooms are believed to offer some protective effect against hospital-acquired infections, including central line-associated bloodstream infections. Yet a recent meta-analysis found the evidence-base to be lacking from a policy perspective. We sought to determine whether private rooms were associated with a lower risk of central-line infections. We examined the discharge records of more than one million inpatients from 335 Texas hospitals to determine patients that stayed in private rooms. Patients who stayed in bay rooms had 64 percent more central line infections than patients who stayed in private rooms. Even after adjusting for relevant covariates, patients assigned to bay rooms had a 21 percent greater relative risk of a central line infection (p = 0.005), compared with patients assigned to private rooms. At the hospital level, a 10% increase in private rooms was associated with an 8.6% decrease in central line infections (p<0.001), regardless of individual patients' room assignment. This study demonstrates and validates the use of private rooms as a structural measure and independent predictor of hospital quality.

## Introduction

Each year, more than 700,000 people contract hospital-acquired infections (HAIs), making this one of the leading causes of preventable medical errors. The most costly and dangerous type of HAI is the central line associated bloodstream infection (CLABSI), which affects about 41,000 people annually [1]. The mortality rate due to CLABSI is 12–25 percent, which corresponds to about 5,000–10,000 preventable deaths per year [2]. Moreover, CLABSIs are estimated to add almost $2 billion to annual health care spending [1].

The purpose of this study is to estimate the protective effect of private hospital rooms for lowering the risk of a CLABSI. We will explore the potential benefits of private rooms at both the hospital-level (percentage of private rooms in a given facility) and the patient-level (assignment to a private room). We shall use the following terminology throughout the article. *Private rooms* are single-occupancy rooms and include both isolation rooms and non-isolation rooms. *Isolation rooms* typically require special ventilation systems and other features to achieve negative air pressure in order to prevent the transmission of pathogens (e.g., Methicillin-resistant Staphylococcus aureus (MRSA)). *Bay rooms*, also called "semi-private" rooms, may contain up to four beds that are typically separated by curtains.

Private hospital rooms have been touted as a means of reducing the risk of an HAI. However, few studies have established a definitive link between hospital design features, including private rooms, and a reduction in HAIs [3]. In 2013, the *Health Environments Research and Design* (HERD) Journal had a special issue on the role of facility design in the prevention of HAIs. According to an editorial in this issue: “There is little evidence.. linking design strategies.. to a reduction in infection rates, causing the experts to question the accuracy of the term “evidence-based design”” [4]. With respect to the built environment, some researchers have recently focused on the importance of cleaning and disinfection of patient rooms for reducing HAIs. One study found that assignment to a hospital room that was previously occupied by a MRSA carrier increased the relative risk of a MRSA infection by 30 percent [5]. Zuberi argued that the outsourcing of housekeeping staff has led to unsafe conditions due to substandard cleaning practices [6].

The structure-process-outcome (SPO) model, as first proposed by Donabedian, provides the conceptual framework for this study [7,8]. Structural characteristics are those stable elements that are necessary, but not sufficient, to achieve excellent health outcomes. Process measures may also include organizational attributes that are fluid and dynamic, such as leadership and communication [9].

“Private rooms” can be considered as either a structure or process measure. Process measures involve daily decisions made by physicians or nurses, such as assigning patients to beds. Structural measures involve decisions made by hospital CEOs in consultation with architects and other professionals. These strategic decisions pertain to the design of new hospitals or the renovation of existing facilities.

Though the literature on private hospital rooms and infection risk is voluminous, the evidence-base remains weak. Dettenkofer et al. conducted a meta-analysis of 178 articles and classified the majority of these at the lowest level of evidence (expert judgment or consensus statements) [10]. Some studies reported a reduction in HAIs after an intervention, such as moving to a new hospital with private rooms. Yet the results could not be generalized due to their small sample sizes and potential confounding bias. A more recent meta-analysis of 25 studies reported similar findings and noted the lack of any randomized, controlled trials (RCTs) [11]. The lack of multisite studies also limits the extent to which these findings can support managerial or policy recommendations [12].

Consider five representative studies from the recent literature on private rooms and HAIs which are summarized in Table 1 [13–17]. Only three of the five studies concluded that private rooms reduced HAIs [14,16,17], while the other two offered more nuanced conclusions [13,15]. In terms of sample size, the studies included at most two hospitals, no more than 49 beds, and a maximum of 19,343 patients. Moreover, none of the studies were performed in US hospitals. By contrast, the present study includes 335 hospitals, more than 90,000 beds, and over one million patients. By including a cross-section of hospitals, our aim is to understand the broader context of private hospital rooms, including organizational factors, patient characteristics, and environmental factors.

**Table 1:** Five previous studies of private rooms and hospital-acquired infections.

Studies [13–17]	Year	Country	Patients	Beds	Findings
Cepeda et al.	2005	England	866	28	HAI not reduced
Bracco et al.	2007	Canada	2,522	14	HAI reduced
Teltch et al.	2011	Canada	19,343	49	HAI reduced
Levin et al.	2011	Israel	210	12	HAI reduced
Ellison et al.	2014	Canada	1,687	35	HAI not reduced

This paper contributes to the current literature in two ways: 1) this study aims to demonstrate, validate, and extend the use of private rooms as a measure of the built environment in health services research studies; 2) The study will show that private hospital rooms were associated with lower CLABSI rates, even after adjusting for patient comorbidities and other characteristics.

By expanding the scope of analysis from one or two hospitals to hundreds of facilities, a broader perspective is sought that may reveal the interaction of private rooms with numerous other organizational attributes, such as hospital ownership, nurse staffing, and teaching status. To determine the potential impact of private rooms, we also sought to better understand the process by which patients are assigned to either a private room or a bay room.

A major obstacle to the use of private rooms as a structural measure has been the lack of available data in secondary datasets. The state of Texas hospital inpatient database includes "hospital room charges" at the patient level that are disaggregated by room type [18]. In contrast to previous studies, the present study disaggregates private rooms into "regular" private rooms, henceforth called "single rooms," and isolation rooms, which employ various design features to prevent the spread of pathogens.

Our dependent variable measuring central-line infections was derived from Patient Safety Indicator (PSI) # 7 [19]. The PSIs were originally developed by the Agency for Healthcare Research and Quality (AHRQ) in order to identify preventable complications. Their development, validation, and ongoing refinements have been described in detail elsewhere [20,21]. This metric has been used in numerous research studies and has been found to have good criterion validity and stability over time [21,22]. However, some have argued that this metric should not be used for public reporting or pay-for-performance due to its relatively low concordance with CLABSI cases identified by chart review [23,24].

## Materials and methods

The data for this study came from the 2013 Texas Inpatient Public Use Data File (TIPUDF), an administrative data set maintained by the Texas Department of State Health Services (TDSHS) [18]. The state database contained one principal and twenty-four secondary diagnosis codes for each discharge and included 93% to 97% of all hospital discharges in the state. Hospital charge data indicated the patient's room type (private, bay (called "semi-private"), ward, and intensive care unit (ICU).) (A more detailed explanation of how hospital room type was defined can be found in the Appendix.) Hospital-level attributes and organizational characteristics were obtained from the 2013 survey of the American Hospital Association (AHA). The study included 335 short-term, acute-care hospitals including specialty hospitals, such as cardiac, surgical, and orthopedic hospitals. Hospitals with less than 25 beds and children's hospitals were excluded.

Consistent with the inclusion criteria of PSI # 7 (Central Venous Catheter-related Bloodstream Infections), the following patients were included in the study: medical and surgical patients age eighteen and older, with a length of stay of two or more days. Patients with a diagnosis of cancer (metastatic cancer, solid tumor, and lymphoma) or HIV were excluded. Patients with a principal ICD-9-CM diagnosis code of 999.31 (infection due to central venous catheter) or 996.62 (Infection due to other vascular device, implant, and graft) were excluded, as this suggested that the infection was present on admission. Patients with a CLABSI that was not present-on-admission were those with a secondary diagnosis code of 999.31or 996.62 [19]. A total of 1,014,903 patients met the inclusion criteria for this study.

Comorbidities, such as congestive heart failure and diabetes, were identified using the Elixhauser comorbidity index, an index that includes 30 comorbidity measures [25]. Comorbidities, such as weight loss and electrolyte imbalance, have been found to increase the risk of a CLABSI [22]. Four comorbidities, cancer (metastatic cancer, solid tumor, and lymphoma) and HIV, were excluded, as stated above. We used the Healthcare Cost and Utilization Project’s Clinical Classifications Software to obtain the most current definitions of the comorbidities [26]. Another important risk factor for CLABSI is whether the patient required renal dialysis [27]. Dialysis patients were identified using the following ICD9CM diagnosis codes: V45.1, V45.11, and V45.12. Less than three percent (n = 14,113) of all patients assigned to private rooms were classified as “private room medically necessary.” We used this as a proxy for patients who were assigned to isolation rooms, since assignment to an isolation room is based on medical exigency.

Hospitals were grouped into two categories based on their predominant room type. Group 1 hospitals (n = 218) were those with at least 50% private rooms. Group 2 hospitals (n = 117) had more than 50% bay rooms. These groups were compared with respect to hospital characteristics (number of beds, nurses-per-bed, teaching status, rural location, and ownership) and patient characteristics (race/ethnicity, gender, number of comorbidities, length of stay, renal dialysis (%), and intra-hospital transfers (%).) Significant differences between the groups were identified using t-tests for ordinal variables and chi-square tests for categorical variables. Unadjusted CLABSI rates per 100,000 patients were calculated for group 1 and group 2 hospitals. CLABSI rates were also calculated by hospital room type based on a pooled sample. Wilson's method was used to calculate the confidence intervals for rare events [28].

Patient-level risk-adjustment models for CLABSI were developed using logistic regression. Predictor variables included both patient characteristics (race/ethnicity, age, sex, dialysis, comorbidities (sum of)) and hospital characteristics (number of beds, nurses-per-bed, and teaching hospital). The patient assignment model (model 1) included hospital room type (private, bay, or isolation) to test the effect of room assignment on CLABSI risk. The hospital design model (model 2) included the hospital's percentage of private rooms as a predictor variable. The reason for using two models was to separate the effects of hospital room types arising from: 1) being assigned to a private room in general; and 2) choosing a hospital with predominantly private rooms.

## Results

A total of 1,357 CLABSI cases (134 per 100,000) were identified among the 1,014,903 patients treated at 335 Texas hospitals during 2013. Percentage of private rooms in a given facility was found to vary by hospital ownership. Non-profit hospitals had the highest median percentage of private rooms (73%), followed by for-profit hospitals (45%), teaching hospitals (44%), and public hospitals (17%). Non-Hispanic white patients were most likely to be assigned to a private room (74%), followed by Asians (66%), Hispanics (62%), and African-Americans (57%).

Table 2 provides a comparison of Group 1 (private room) and Group 2 (bay room) hospitals with respect to patient and hospital characteristics. Group 2 hospitals were more likely to have for-profit or public ownership. Group 2 hospitals also treated more Hispanics, African-Americans, and fewer women. They also had 30% fewer nurses-per-bed (1.10 vs. 1.56; p < 0.001) and more patients who required renal dialysis (4.0% vs. 3.3%; p<0.001). Patients from Group 2 hospitals had a longer length of stay (5.6 vs. 4.8; p<0.001) and more intra-hospital transfers (15.2% vs. 13.7%; p<0.001). Patients from group 1 hospitals had more comorbidities (3.28 vs. 3.24; p<0.001).

**Table 2:** Comparison of Group 1 (private room) hospitals and Group 2 (bay room) hospitals.

			Group 1	Group 2	
Variable			(n = 218)	(n = 117)	P-value
***Hospital Characteristics***					
	**Beds (licensed)**		230.6	263.3	0.687
	**Nurses-per-Bed (FTE)**		1.6	1.1	<0.001
	**Teaching hospital (%)**		8.9%	10.3%	0.791
	**Rural location (%)**		18.2%	21.4%	0.871
	**Ownership (%)**				<0.001
		Non-profit	47.1%	23.6%	
		Public ownership	6.7%	15.5%	
		For-profit	46.2%	60.9%	
***Patient Characteristics***					
	**Number of Patients**		693,966	320,937	
	**Comorbidities, mean**		3.3	3.2	<0.001
	**Length of stay (days)**		4.8	5.6	<0.001
	**Dialysis rate (%)**		3.3%	4.0%	<0.001
	**Intra-hospital Transfers**				<0.001
		Zero	86.3%	84.8%	
		One or more	13.7%	15.2%	
	**Race/Ethnicity (%)**				<0.001
		Non-hispanic White	65.0%	46.9%	
		Non-hispanic African-American	9.5%	11.9%	
		Asian	1.3%	1.7%	
		Hispanic	20.5%	33.8%	
		Other	3.7%	5.8%	
	**Female (%)**		57.5%	55.2%	<0.001

**Fig 1:**
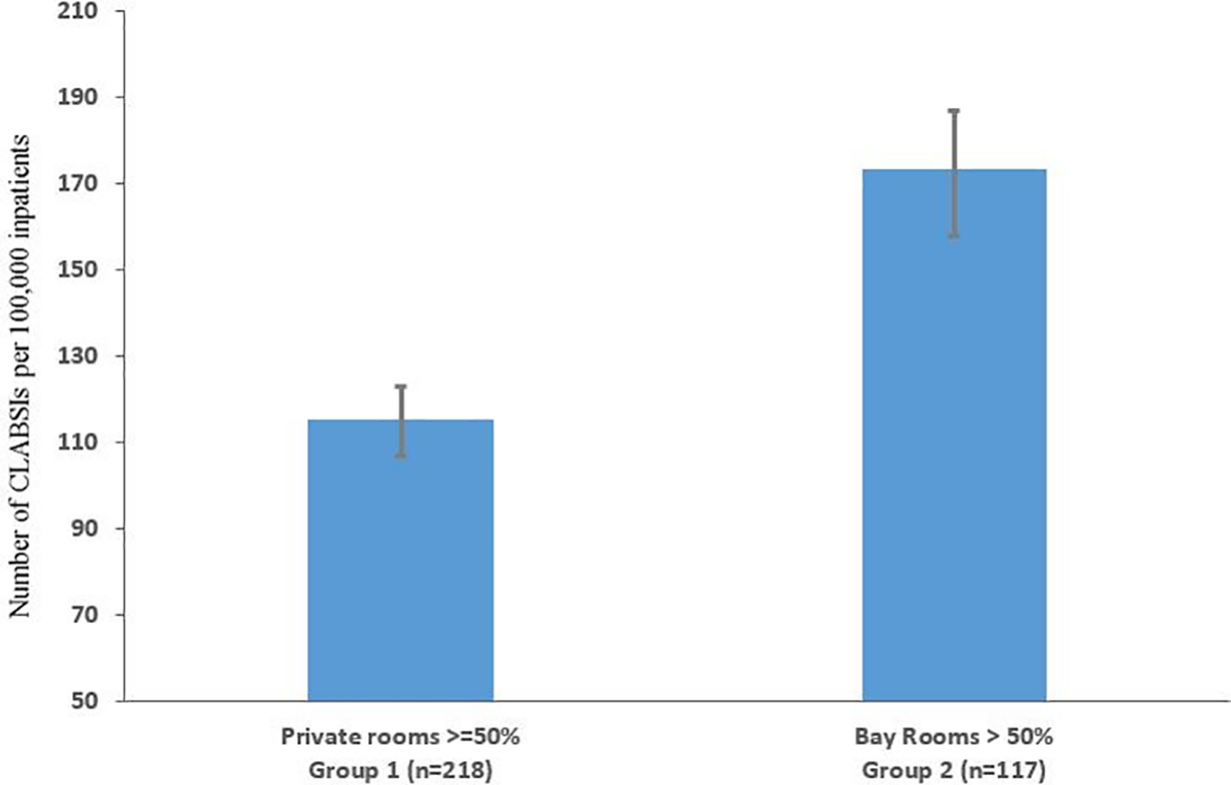
Rate of central-line bloodstream infections at 335 Texas hospitals. Group 1 Hospitals have majority private rooms. Group 2 Hospitals have majority bay rooms.

As shown in Fig 1, Group 1 hospitals had 33 percent fewer CLABSIs overall than Group 2 hospitals (115 vs. 173; P<0.001). Fifteen percent of Group 1 hospitals reported having zero CLABSIs compared with only two percent of Group 2 hospitals (P<0.001). Mortality rates among CLABSI patients were significantly higher in Group 2 compared to Group 1 (7.9% vs. 4.4%; P<0.001). Hence the risk of mortality due to CLABSI was more than twice as high in Group 2 hospitals. Among patients assigned to private rooms, patients from Group 1 had 27 percent fewer CLABSIs than patients from Group 2 (100 vs. 136; p<0.001).

**Fig 2:**
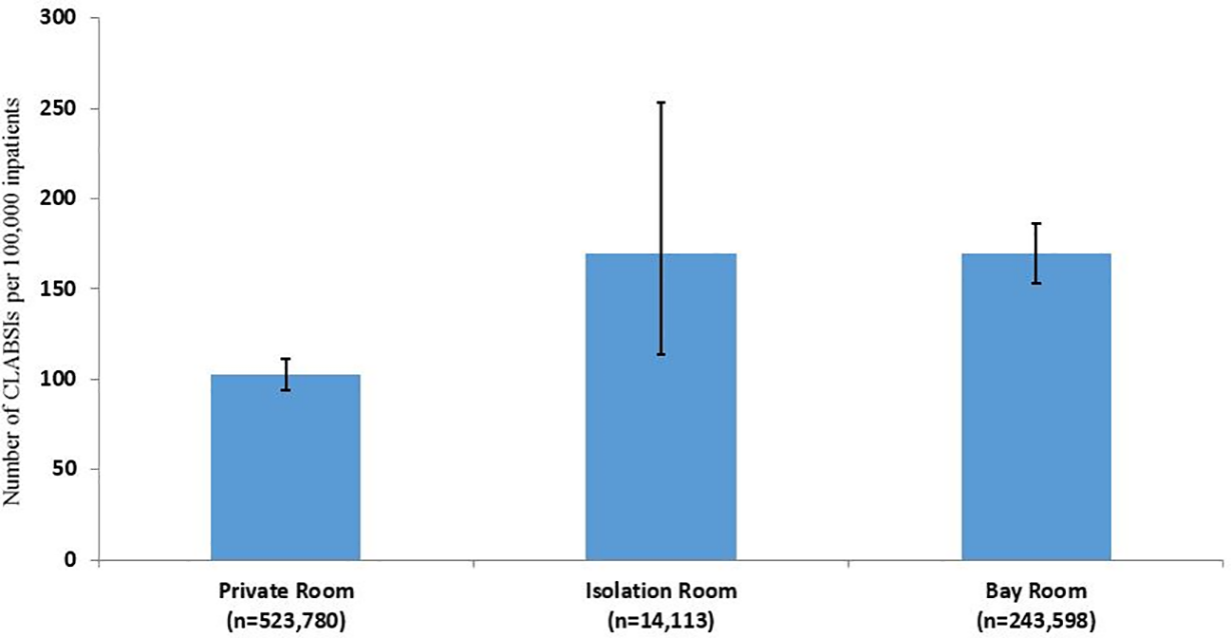
CLABSI Rates based on type of hospital room where patient stayed.

Based on the pooled results shown in Fig 2, patients who stayed in bay rooms had 64% more CLABSIs per 100,000 than patients who stayed in private rooms (169 vs. 103; p<0.001). There was no significant difference in CLABSI rates between patients assigned to bay rooms compared to isolation rooms (169 vs. 170; p>0.05.)

As shown in Tables 3 and 4, a higher risk of CLABSI was associated with being African-American, Hispanic, male, having more comorbidities, and requiring kidney dialysis. CLABSI risk was also higher for teaching hospitals and those with fewer nurses-per-bed. Lower CLABSI risk was associated with being female, being non-Hispanic white, increased age, fewer comorbidities, and more nurses-per-bed.

**Table 3:** Impact of room assignment and other variables on CLABSI risk.

			Adjusted Odds Ratio	P-value
Variable			(95% CI)	
***Patient Factors***				
	**Room Type Assigned**			
		***Baseline*: *Private Room***	—	—
		**Bay Room**	1.212 (1.060–1.385)	0.005
		**Isolation Room**	0.648 (0.425–0.989)	0.044
	**Age**		0.976 (0.972–0.979)	<0.001
	**Female**		0.578 (0.508–0.658)	<0.001
	**Race/Ethnicity**			
		*Baseline*: *White*, *non-Hispanic*	—	—
		African-American, non-Hispanic	6.348 (5.345–7.538)	<0.001
		Asian	0.669 (0.249–1.799)	0.426
		Hispanic	1.397 (1.163–1.679)	<0.001
		Other/Unknown	7.136 (5.794–8.788)	<0.001
	**Comorbidity**		1.386 (1.347–1.426)	<0.001
	**Dialysis**		2.868 (2.422–3.396)	<0.001
***Hospital Characteristics***				
	**Beds (in 00s)**		0.955 (0.935–0.976)	<0.001
	**Nurses-per-Bed**		0.493 (0.431–0.565)	<0.001
	**Teaching hospital**		2.021 (1.710–2.388)	<0.001

**Table 4:** Impact of hospital private room (%) on CLABSI risk.

			Adjusted Odds Ratio	P-value
Variable			(95% CI)	
***Patient Factors***				
	**Age**		0.976 (0.972–0.980)	<0.001
	**Female**		0.589 (0.518–0.670)	<0.001
	**Race/Ethnicity**			
		*Baseline*: *White*, *non-Hispanic*		—
		African-American, non-Hispanic	5.934 (5.004–7.038)	<0.001
		Asian	0.631 (0.235–1.695)	0.361
		Hispanic	1.378 (1.148–1.655)	0.001
		Other/Unknown	6.417 (5.212–7.902)	<0.001
	**Comorbidity**		1.391 (1.352–1.431)	<0.001
	**Dialysis**		2.894 (2.444–3.427)	<0.001
***Hospital Characteristics***				
	**Private Rooms (%)**		0.991 (0.989–0.993)	<0.001
	**Beds (in 00s)**		0.953 (0.933–0.974)	<0.001
	**Nurses-per-Bed**		0.49 (0.428–0.561)	<0.001
	**Teaching hospital**		2.005 (1.703–2.360)	<0.001

After adjusting for relevant covariates, model 1 results show that patients who were assigned to a bay room were 1.21 times (CI: 1.06, 1.38; p = 0.005) more likely to develop a CLABSI, compared with patients assigned to a private room. Whereas isolation rooms may offer some protective benefit, the odds ratio of 0.648 was inconclusive, as it was not significant at the one-percent level (p = 0.044).

As shown in Table 4, a higher percentage of private rooms in a given hospital was associated with a lower risk of CLABSI for all patients, regardless of room assignment. Hence patients could potentially reduce their CLABSI risk simply by choosing a hospital with a high percentage of private rooms. Based on the odds ratio of 0.991 for private room (%), a hospital could reduce its CLABSI infections by about 8.6% for every 10% increase in private rooms. These results indicate that hospitals with mostly private rooms may have some protective effect or "positive externality" that benefits all hospital patients, including those assigned to bay rooms.

## Discussion

We found a positive association between the percentage of bay rooms in a given hospital and CLABSI rates, even after adjusting for patient risk factors and other variables. Among patients who stayed in Group 2 hospitals, those who were assigned to private rooms had a 24% relative risk reduction for CLABSI compared to those who stayed in bay rooms. These findings are consistent with case study reports on the best practices for reducing CLABSIs, which include moving to an all-private room design [29]. Though often yielding important insights, previous studies were constrained by small sample sizes and potential confounders. By contrast, the present study included over one million patients and 335 acute-care hospitals from a single state.

This study also found that hospitals with mostly private rooms, that is, Group 1 hospitals, also had a shorter length of stay, fewer intra-hospital transfers, and higher nurse staffing levels than Group 2 hospitals. Each of these has also been associated with fewer HAIs and lower CLABSI rates. They also suggest the various pathways by which private rooms can facilitate better outcomes. Private rooms have also been found to foster improved communication and coordination among clinical staff [30].

Nurse staffing was found to play an important role in reducing CLABSI rates, and this finding is consistent with four previous studies [31–34]. Group 1 hospitals had 42% more nurses per bed than Group 2 hospitals, which partly reflects the added nurse staffing requirements of the private-room design [31]. "Nurses-per-bed" was also a significant predictor of CLABSI risk. Based on the results of the logistic model in Tables 3 and 4, a 10% increase in nurses per bed was associated with a 6.7% decrease in a hospital's CLABSI rate.

Of all the patients assigned to some type of private room, less than three percent of patients (n = 14,113) were assigned to a private room due to "medical necessity." These patients had more comorbidities (3.6 vs. 3.0; p<0.001) and were twice as likely to require hemodialysis (6% vs. 3%; p<0.001) compared to patients assigned to regular private rooms. That is, these patients had multiple risk factors for CLABSI. After adjusting for these factors, CLABSI rates for patients in isolation rooms were not measurably different than those assigned to (non-isolation) private rooms. Hence there was no clear evidence that isolation rooms provided any additional protective effect, beyond that of a regular private room. This is consistent with two previous studies [13,35]. Cepeda *et al*. argued that any potential benefit from transferring a patient to an isolation room must be weighed against the risk from the transfer itself [13]. Another study found that patients in isolation had less frequent contact with nurses [35].

We also found significant differences in the likelihood of being assigned to a private room by race/ethnicity. Compared with non-Hispanic whites, African-Americans were 1.63 times more likely and Hispanics were 1.44 times more likely to stay in a bay room. Most of these differences appear to be driven by the hospital's geographic location.

Our study has several limitations. Because our data come from a single state, caution is warranted in generalizing our findings to other hospital populations. Texas has a diverse population, geographic diversity, population growth, and numerous hospitals. It is also one of thirteen states that does not have certificate-of-need (CON) laws that may limit the building of new hospitals [36]. Texas is also one of 27 states that mandate public reporting of CLABSI rates [37]. In spite of this limitation, we believe that our study represents an advance over previous small-sample studies and provides a basis for further research in this area.

Finally, we acknowledge the limits of our measure of CLABSIs, which is one of the patient safety indicators. While the PSIs have been used in health services research studies since 2003, their use remains controversial, especially as it relates to pay-for-performance and public reporting [23,38]. However, each approach for identifying patient safety events is subject to criticism, regardless of whether such measures are derived from discharge abstract databases or from patient chart abstracts [12]. Although much progress has been made in recent years, there remains a need for better documentation of CLABSIs and other HAIs, as well as standardized data collection mechanisms [37]. According to Rivard, Rosen, and Carroll, PSIs are best used as higher-level measures of patient safety rather than as definitive indicators of preventable events [39].

The primary advantage of including data on more than one million patients is that it can yield insights into the "epidemiology" of private hospital rooms, as well as the process by which patients are "sorted" into bay rooms and private rooms. Moreover, these results show the interdependence of private rooms with other important organizational factors that constitute the broader environment in which care is delivered.

Since 2006, private rooms have become the predominant room type for new hospital construction within the US, and the prevalence of bay rooms has declined over time [30]. The reasons for this are complex and well-documented elsewhere [40,41]. According to our findings, the bay room is still the most prevalent room type for public hospitals and within certain hospital markets, such as those in the southern and western regions of the state.

The rise of the private hospital room has coincided with many other changes in the built environment, such as decentralized nursing stations, acuity-adaptable rooms [42], use of antimicrobial surfaces, and improved air filtration [4,30]. Many experts believe that the cumulative impact of these design changes that are associated with modern facilities has created a safer hospital and a better healing environment [4,30,41]. These same experts also concede that there are few well-designed studies linking changes in the physical environment to better clinical outcomes and a reduction in HAIs [4,30].

According to a recent CDC report, the incidence of HAIs has decreased significantly since 2008, and CLABSI rates have fallen by about 50 percent [43]. While the main causes for this have been well-documented elsewhere [44], it is likely that changes in the built environment and improvements in hospital design have also played a significant role.

Due to data limitations, it was not possible to separate the protective effect of private rooms from that of other hospital design trends mentioned earlier. Thus, private rooms may also be a proxy variable for a newer hospital with a more modern design. Even within a given hospital, design features and physical layout may vary by floor or unit, especially for older facilities that have been renovated or expanded. To further validate our methodology and findings, we collaborated with a nearby safety-net hospital and presented our recommendations on the benefits of the all private-room design for a planned hospital expansion project. We also examined whether private rooms and bay rooms within this facility differed with respect to other design features such as sink placement, acuity-adaptable beds, and air filtration systems.

Finally, this study focused on only one potential benefit of private rooms, that is, their protective effect for reducing the number of CLABSIs and other HAIs. Yet there are numerous other potential benefits of private rooms that warrant further study. These include: improved patient privacy, reduced medical errors, improved nurse satisfaction, reduced noise, improved sleep, and reduced length of stay [31]. Future research should extend this analysis to include a broader scope of process and outcome measures, such as measures of patient and employee satisfaction.

## Supporting information

S1 Appendix Estimating the percentage of private rooms for each hospital. (DOCX)
