# Supplementary material for: The role of the built environment and private rooms for reducing central line-associated bloodstream infections
Source: PLoS One. 2018 Jul 27;13(7):e0201002. doi: 10.1371/journal.pone.0201002 (PMC6063409; doi:10.1371/journal.pone.0201002)
Supplement: S1 Appendix — (DOCX) [file pone.0201002.s001.docx]

**S1 Appendix:**

Estimating the "Percentage of Private Rooms" for each Hospital

Texas hospital inpatient data contained hospital charges. Room charges included several variables, such as private room, bay room (called "semi-private"), ward, and intensive care unit (ICU). Examining the percentage of patients who stayed in private rooms relative to bay rooms allowed us to make some inferences regarding the hospital's physical layout. However, there were several complicating factors and potential sources of bias that had to be taken into account. For example, an intra-hospital transfer occurs when a patient is transferred to a different bed in the same hospital. About 14% of the patients in our sample stayed in more than one type of room, even as this is a lower bound on the true number of intra-hospital transfers.

Whether a given patient is assigned to a private room depends on many factors, such as the patient's insurance, diagnosis, and the hospital's physical design. Suppose that 70% of a hospital's acute-care beds are in private rooms. Given a sufficient sample, we would expect about 70% of patients to be assigned to private rooms. However, if “bed assignment rules” give higher priority to private rooms, then the *effective* rate of private room assignment would be higher than predicted based on the hospital's physical layout.

For the purposes of infection control, the relevant metric is the percentage of patients who are assigned to a private room and remain in a private room throughout their hospital stay. Thus, the numerator for each hospital consisted of the "number of patients assigned to a private room," as defined below. The denominator consisted of the number of patients assigned to an acute-care bed in either a private or a bay room. (Note there are few remaining “ward rooms” in modern hospitals.) Thus, (Private room %) = 1 - (Bay room %).

Adult patients (age 18+) who spent at least one day in a bay room were assigned to that category. Other (acute-care) patients who spent at least one day in a private room were classified as “private room.” (Note that we did not have information on whether ICU beds were located in private rooms. Twenty-three percent of all inpatients were not acute-care patients. Hence, they were not assigned to either category (bay or private). This explains the differing sample sizes in Figs 1 & 2.)
